# Estimation of the cost-effective threshold of a quality-adjusted life year in China based on the value of statistical life

**DOI:** 10.1007/s10198-021-01384-z

**Published:** 2021-10-16

**Authors:** Dan Cai, Si Shi, Shan Jiang, Lei Si, Jing Wu, Yawen Jiang

**Affiliations:** 1 School of Public Health (Shenzhen), Sun Yat-Sen University, Shenzhen, Guangdong China; 2 School of Population and Public Health, University of British Columbia, Vancouver, BC Canada; 3 The George Institute for Global Health, UNSW Sydney, Kensington, Australia; 4 School of Health Policy and Management, Nanjing Medical University, Nanjing, Jiangsu China; 5 School of Pharmaceutical Science and Technology, Tianjin University, No. 92 Weijin Road, Nankai District, Tianjin, China

**Keywords:** Willingness-to-pay, QALY, Threshold, China, I18, I18

## Abstract

Cost-effective threshold (CET) is essential for health technology assessment and decision-making based on health economic evaluations. Recently, it has been argued that the commonly used once and three times of gross domestic product (GDP) per capita CETs of a quality-adjusted life year (QALY) are not necessarily empirically supported in all countries. Therefore, we aimed to estimate the CET of a QALY as times of GDP per capita in China, of which the reimbursement coverage decisions are increasingly engaging economic evaluations. Estimates on the value of statistical life (VSL) in China were identified from several studies in the literature and converted to times of GDP per capita, the weighted average of which was used for subsequent calculation. By pooling data on population mortality, health utility, and age distribution, we estimated the value of a statistical QALY (VSQ) from VSL using an established mathematical process, which represented the theoretical upper bound of CET. The corresponding point estimate and theoretical lower bound were obtained using their numerical relationships with the upper bound. Scenarios analyses were also conducted. The estimated CET, its upper bound, and its lower bound were 1.45, 2.90, and 1.16 times of GDP per capita in China, respectively. In different scenarios, the estimated CET varied but was greater than once GDP per capita in most cases. As such, the CET of a QALY in China is close to 1.5 times of GDP per capita, which should be benchmarked for future ICER-based coverage decisions.

**Supplementary Information:**

The online version contains supplementary material available at 10.1007/s10198-021-01384-z.

## Background

Commonly used in drug reimbursement policymaking and designs of vaccination programs, cost-effectiveness analysis (CEA) is an important tool to inform healthcare decisions [1]. Globally, there is a spectrum of weights that CEA carries in decision-making ranging from complete absence to heavy reliance. Although increasingly popular as an auxiliary tool for price negotiation, CEA does not have a formal role in public or private financing decisions except for immunization programs [2] in fragmented and decentralized healthcare systems such as the United States (US). Unlike the US, CEA is used to guide price-setting in markets like Japan and Germany [3, 4]. In the rare case of England and Wales, CEA is mandatory to be considered for reimbursement [5]. In China, CEA has received growing attention in the drug benefit design process of the National Healthcare Security Administration (NHSA) in recent years, although it is not yet a compulsory part of the reimbursement negotiation documents [6]. In fact, the prevailing consensus of the administrative and academic communities is that CEA will be an integral part of the appraisal process [6–9]. In light of such a momentum, it is critical to set up the decision rules based on CEA results, an indispensable step of which is an explicit cost-effective threshold (CET).

CET quantifies the marginal efficiency of interventions within the healthcare system and represents the efficiency frontier of the healthcare system under a constrained budget. Theoretically, all interventions with incremental cost-effectiveness ratios (ICERs) below the CET should be adopted to minimize opportunity costs. The threshold of once and three times gross domestic product (GDP) per capita for each quality-adjusted life year (QALY) gained are commonly cited in the literature [10]. However, these rules have neither solid theoretical basis nor convincing empirical evidence [10, 11].

Driven by the mounting CEA-based decisions globally, the literature on estimating the CET has been reviving in the health economics community. To that end, at least four possible approaches have been proposed. First, the implicit CET can be determined by reviewing previous decisions and identifying the point beyond which public financing is rejected [5]. However, this approach is not always feasible, because it relies on the availability of the economic evaluations of all interventions and the assumption that the evaluations can reflect the true cost-effectiveness profiles. Second, the CET can be measured by willingness-to-pay (WTP), which is elicited using either stated or revealed preference [12, 13]. The WTP-based estimate provides a demand-side threshold of the value of a QALY from the consumer perspective. Consistent with the welfarist theories, the demand-side value of a QALY is a good reference for decisions on healthcare budget expansion by drawing resources from other sectors, yet it is not equally relevant in determining the trade-offs given a specific healthcare budget [14, 15]. Third, the threshold may be estimated by measuring the QALY gain corresponding to marginal increases in healthcare expenditures. However, the identification of the impact of budget increases on QALYs is contingent upon the existence of strongly exogenous healthcare expenditure shocks. Using routinely collected costs and health outcome data ignore the contribution to health from other sectors including but not limited to education, environment, transportation, and housing. The exogenous budget shock should also endure long enough for minimally detectable health consequences to occur at the population level. A good example was a study by Vallejo‐Torres et al. that estimated the CET in Spain by exploiting exogenous healthcare budget plummeting due to the economic crisis in 2008 [16]. Such an approach is not always replicable, since exogenous variations in healthcare expenditure are rare. Fourth, the CET has been estimated using the value of statistical life (VSL) approach, in which the values of QALYs are mathematically linked to VSL. Although relatively viable and straightforward, this approach requires solid VSL estimates. With each of the methods possessing its own strengths and weaknesses, there are no universally agreed-upon approaches. In this analysis, we aimed to analyze the CET in China using the VSL approach, which quantifies CET through the value of a statistical QALY (VSQ). The VSL approach was preferable for several empirical and theoretical reasons. To the extent that the present study aimed to evaluate the CET of the healthcare system within its own budget constraint, stated-preference estimates of WTP are not necessarily the optimum approach. Also, exogenous healthcare expenditure shocks similar to the 2008 economic crisis that affected Spain was not available in China. More, the approach of using prior coverage decisions, although technically feasible, would still likely fail to generate reliable CET estimates, because CEA evidence directly pertaining to the coverage decisions was not available. Specifically, the CEA data that the Chinese authority used to make decisions were unknown, which hampered reliable estimation using prior coverage decisions.

Evidence on the CET of a QALY of the Chinese population is absent. Therefore, the present study contributes important information by filling this void. An estimate of the CET of a QALY provides a rationale for scientifically rigorous healthcare decision-making [17]. In light of this, it is recommended for each country to develop its own CET to enable the alignment of clinical and economic value with the efficiency frontier under a fixed budget within each healthcare system [18], which is of fundamental importance for China that is facing increasing pressure of healthcare expenditure [19].

## Literature review

To take an audition of the current progresses in the estimates of CET per QALY for the Chinese population, we searched for studies that contained (“willingness-to-pay” OR “WTP” OR “value” OR “cost-effective threshold” OR “CET”) AND “China” AND (“QALY” OR “quality-adjusted life year”) in the title or abstract. The time span specified for the searching was database inception through July 2021. To be included, studies had to have explicitly estimated the threshold. We also excluded studies that were not reported as full-length articles. Using these criteria, none of the studies in the literature attempted to estimate the CET of a QALY for the Chinese population by far.

Internationally, explicit estimates of the CET of a QALY are not available in most countries. The leading practice in the past decades was to rely on the arguably arbitrary once and three times the GDP per capita thresholds [10]. Realizing the potential drawbacks of these arbitrary quantifications, economists in several countries have taken the initiative to refresh the CET estimates. For example, Lankarani et al. estimated the value of the general population’s WTP for a QALY in Iran [20]. They showed that the WTP for a QALY was US$2,847 in 2017, which was equivalent to 0.57 of Iranian GDP per capita. Recently, Vanness et al. estimated the CET in the US based on health opportunity costs [21]. According to their estimates, the CET per QALY in the US was US$104,000 in 2019, which was about 1.5 times the GDP per capita of the US in the same year. More, Tehard et al. estimated the CET in France to be €147,093 using the VSL method [12]. This threshold was slightly over 3.5 times the GDP per capita of France at the time of estimation.

The rapid growth of country-specific CET estimates suggests a substantial unmet need of related evidence in the current literature, which we aimed to document for the Chinese population in the present study. On top of the evidentiary contribution, we also introduced marginal novelty of CET estimation process by combining an established VSL-based approach and recent advances in research on adjusting VSL-based threshold estimates.

## Methods

### Estimation process

The estimation approach in the study conducted by Tehard et al. for VSQ in France was adopted with a revision in the present analysis [12]. To elaborate the estimation of VSQ based on VSL, it is important to introduce the estimation of the value of a life year (VoLY) first

1 $${\text{VSL}} = \mathop \sum \limits_{i = 1}^{T\left( a \right)} {\text{VoLY}}\left( a \right) \times \left( {1 + r} \right)^{{ - \left( {i - 1} \right),}}$$

in which *T*(*a*) is the life expectancy at age *a*, *r* is the yearly discount rate, VoLY(*a*) is the average value of a life year for the remaining life years at age *a*, and *i* is the *i*th year in the remaining life expectancy starting from age *a*. VoLY(*a*) takes the same constant value for all life years over the life expectancy of an individual at age *a*. Since VoLY(*a*) is a constant value, Eq. (1) can be transformed to

2 $${\text{VSL}} = {\text{VoLY}}\left( a \right)\mathop \sum \limits_{i = 1}^{T\left( a \right)} \left( {1 + r} \right)^{{ - \left( {i - 1} \right)}}$$

The following equivalence can be derived from Eq. (2):

3 $${\text{VoLY}}\left( a \right) = \frac{{{\text{VSL}}}}{{\mathop \sum \nolimits_{i = 1}^{T\left( a \right)} \left( {1 + r} \right)^{{ - \left( {i - 1} \right)}} }}$$

In addition to the importance of VoLY in its own rights, another importance of Eqs. (1)–(3) is that a similar relationship can be established for VSQ. For a given individual, QALYs in the remaining life years are the sum of weighted remaining life years, the weights of which are the utility values representing the quality of life of health states during each time period. To illustrate, we begin with the relatively straightforward relationship

4 $${\text{QALYs}} = \mathop \sum \limits_{i = 1}^{T\left( a \right)} u_{i} ,$$

where *u*_*i*_ is the health state utility of the *i*th year in the remaining *T*(*a*) life years starting from age *a*. Similar to VoLY(*a*), VSQ(*a*) is a constant for a given individual at age *a*. Therefore, it can be shown that

5 $${\text{VSL}} = \mathop \sum \limits_{i = 1}^{T\left( a \right)} {\text{VSQ}}\left( a \right) \times u_{i} \times \left( {1 + r} \right)^{{ - \left( {i - 1} \right)}} = {\text{VSQ}}\left( a \right)\mathop \sum \limits_{i = 1}^{T\left( a \right)} u_{i} \times \left( {1 + r} \right)^{{ - \left( {i - 1} \right)}} ,$$

following which the relationship in Eq. (6) can be established:

6 $${\text{VSQ}}\left( a \right) = \frac{{{\text{VSL}}}}{{\mathop \sum \nolimits_{i = 1}^{T\left( a \right)} u_{i} \times \left( {1 + r} \right)^{{ - \left( {i - 1} \right)}} }}$$

VSL is a fixed number that embodies the aggregate dollar amount the population are collectively willing to pay to reduce the mortality risk regardless of age [22]. The underlying assumption is that all lives are equally valued from the societal perspective [12]. On the other hand, VSQ(*a*) is a function of age by definition. However, the estimate of CET of a QALY mandates a single index VSQ. To obtain such a value, it is necessary to calculate the weighted average value of VSQ(*a*)s across age groups. To that end, the VSQ(*a*) for each age between 0 and 99 years were calculated and then weighted by the population size of the corresponding age group in China.

### Input

To enable the estimation, several input values were required. Specifically, an estimated VSL, the discount rate, age-specific utility values, age-specific mortality data, and population tabulation by age are necessary to calculate VSQ.

**Fig. 1:**
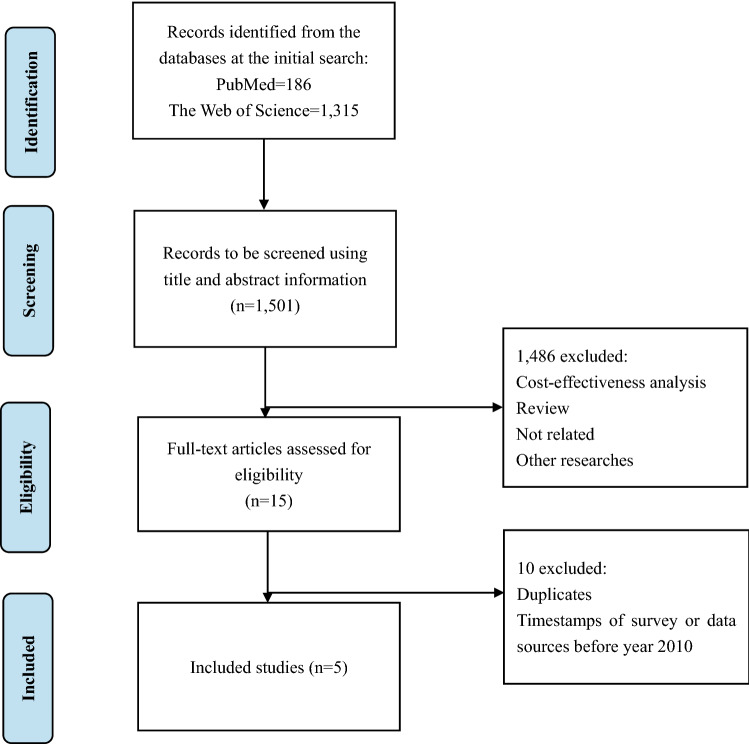
The identification and screening process of value of statistical life studies in China

There are no official VSL estimates in China to date. Therefore, we used studies in the literature that provided Chinese VSL estimates. Specifically, we searched for studies that contained “value of statistical life”, “VSL”, or “vsl” in the title or abstract that also contained “China” in the title or abstract in PubMed and Web of Science databases. In addition, studies that contained both “willingness-to-pay”/“WTP” and “health”/“mortality” in the title or abstract that also contained “China” in the title or abstract were identified initially. Studies had to have explicitly estimated VSLs to be eligible, such that those merely used VSL estimates from other studies for research were excluded. An example of the keywords and search strategies using PubMed are presented in supplementary files Table S1. We excluded studies that were not reported as full-length articles. Studies in which the data were collected before 2010 were also excluded. The list of included studies is given in Table 1. The flowchart of study identification and screening process is displayed in Fig. 1.

**Table 1:** Included VSL studies in China

Author (year) [references]	Survey location	Survey year	Subgroup	Sample	VSL in survey year (CNY)	Local GDP per capita in survey year (CNY)	VSL/GDP per capita
Yang et al. (2016) [38]^a^	Nanjing, China	2014–2015	Motorists	1602	¥3,729,493	¥107,545^b^	32.85
			Non-motorists	1255	¥3,281,283		
Hammitt and Geng (2019) [39]	Chengdu, China	2016	NA	1051	¥3,852,800	¥76,960	50.06
Jin et al. (2018) [40]	Beijing, China	2016	NA	1107	¥5,540,000	¥124,516	44.49
Hao et al. (2019) [41]	74 major Chinese cities	2016	NA	308	¥1,530,000	¥49,178	31.11
Zheng et al. (2019) [42]^a^	Hangzhou, China	2017	Drivers	692	¥3,870,402	¥135,113	27.26
			Non-drivers	400	¥3,359,281		

*NA* not available

^a^In these two studies, the weighted average VSL of subgroups were used

^b^The GDP per capita of Nanjing in 2014 was used

It was noteworthy that most studies we identified were conducted in relatively wealthy regions of China, such that the VSL estimates were unlikely to be representative nationwide. Therefore, we standardized these VSL estimates as times of GDP per capita of each corresponding region in the year of data collection instead of original monetary values, which was a deviation from the method proposed by Tehard et al. [12]. Assuming that the VSL as times of GDP per capita was stable across regions and over the recent years, such processing of VSL estimates both attenuated the bias due to unrepresentativeness of data collection sites and sidestepped the need of inflating monetary values. Accordingly, the final estimates and VSQ were also standardized as times of GDP per capita. To permit such numeric representation, data on GDP per capita of the involved regions were retrieved from the corresponding local statistics yearbooks [23–26]. The weighted mean of the standardized VSQs were calculated for subsequent estimation, the weights of which were the sample sizes in each of the four included VSL studies. To illustrate, the weighted mean of standardized VSQ was calculated as

7 $${\text{The weighted mean of standardized VSQs}} = \mathop \sum \limits_{j}^{J} \frac{{{\text{monetary VSQ}}_{{\text{j}}} }}{{\left( {\text{GDP per capita}} \right)_{{\text{j}}} }} \times \frac{{n_{j} }}{N},$$

among which monetary VSQ*j* is the monetary VSQ of the region in the *j*th study, (GDP per capita)_j_ is the GDP per capita of the *j*th study in the year of VSL data collection, *n*_*j*_ is the sample size in the *j*th study, and *N* is the total sample size of all studies. If two or more regions were surveyed within an individual VSL study, the weighted mean of standardized VSQs was calculated within the study first and then plugged into Eq. (7).

Per the Chinese pharmacoeconomics guidelines, an annual discount rate of 5% was used [27]. Also, the health utility values of the general population by age group were obtained from a nationwide survey using EQ-5D in China except the age group of 14 years and younger [28]. For this age group, the corresponding data were absent. Therefore, it was assumed that they had a health utility value of unity. More, age-group utility values were reported by sex. To accommodate such data reporting to the present analysis, the average utility value of men and women within each age group was computed. Even more, age-specific life expectancy data were estimated using age-specific death probability information from the complete Chinese life table based on the 2010 national whole population census [29]. Similar to life expectancy, the age-defined subpopulation sizes were based on data from the 2010 national whole population census [30]. Whereas a more recent national whole population census was conducted in 2020, data from the newest census have not been published yet. Data on age-specific life expectancy and population distribution are provided in Table S2 (Additional file 1), whereas health utility values are presented in Table S3 (Additional file 1).

### Adjustment using the overestimation factor

Recently, Herrera-Araujo et al. [31] showed that VSQ estimates using the VSL approach represented not necessarily the true CET but rather the upper bounds of CET. Specifically, the CET of health improvement depended on the baseline health status, and the worse the health status is, the greater an individual gains from marginal improvement in health. Since CET represents the amount of wealth people are willing to trade off for health improvement, it is the monetary equivalent of the marginal utility of health change. Formally, the theoretical lower (CET_L_) and upper (CET_U_) bounds of CET are

8 $$\frac{{U\left( {w, \max \left( q \right)} \right) - U\left( {w, q^{*} } \right)}}{{\max \left( q \right) - q^{*} }} \le U^{\prime } \left( {w,q^{*} } \right) \le \frac{{U\left( {w, q^{*} } \right) - U\left( {w, \min \left( q \right)} \right)}}{{q^{*} - \min \left( q \right)}},$$

where $U\left(w, q\right)$ is the utility function with respect to wealth $w$ and health status $q$, $\mathrm{max}\left(q\right)$ is the maximum health status, $\mathrm{min}\left(q\right)$ is the minimum health status that is usually taken as death, ${q}^{*}$ is the baseline health status that respondents implicitly relate to when they try to value life and health, and ${U}^{^{\prime}}\left(w,{q}^{*}\right)$ is the marginal utility of health change. Since VSL elicitation directly relates to mortality risk reduction, it is the narrative counterpart of the upper bound in Eq. (8). In the study, Herrera-Araujo et al. [31] further delineated the spread between CET and its bounds using the functional form of constant relative risk aversion (CRRA). Using empirical measures of risk aversion, the same study also calibrated the parameters of the CRRA function and demonstrated that the VSL approach overestimated the CET by a factor of two on average, whereas the average ratio of the lower bounds of CET to the upper bounds was 0.4 [31]. Using these numeric relationships for adjustment, we estimated the VSQ and its lower bound based on the upper bound from Eq. (7).

### Sensitivity analyses and uncertainty

The results of the present analysis hinged on an array of input parameters, the values of which were retrieved from the literature. Therefore, a number of scenario and sensitivity analyses were conducted.

The first set of scenarios involved using alternative VSL values. Specifically, the individual estimate from each of the four identified studies was used in lieu of their weighted mean. In the second set of scenarios, the age-specific utility value inputs were replaced by scores sourced from two additional studies that investigated the population quality of life in China [32, 33].

To account for uncertainty of utility inputs in a way that resembles deterministic sensitivity analysis, the base-case utility inputs were varied by 10% upward and downward. In addition, the annual discount rate was changed to 3% and 8%, respectively. To estimate the 95% confidence intervals (CIs) of VSQ, Monte Carlo simulations with 1000 repetitions were conducted. In these simulations, utility values followed beta distributions, whereas mortality rates and VSL inputs followed normal distributions. The standard errors of utility values were assumed to be 10% of the means, whereas the standard errors of death probabilities were estimated using

9 $${\text{SE}}_{i} = \sqrt {Q_{i}^{2} \cdot \frac{{1 - Q_{i} }}{{D_{i} }}} ,$$

where $S{E}_{i}$ is the standard error of death probability of age $i$, ${Q}_{i}$ is the death probability of age $i$, and ${D}_{i}$ is the number of deaths of the age $i$ population [34]. Unlike the upper and lower bounds from Eq. (8), the 95% CIs represented uncertainty from the empirical estimation process.

## Results

In the base case, the weighted mean of the standardized VSQs without overestimation adjustment, which was also the theoretical upper bound of CET, was 2.90 times of GDP per capita. Accordingly, the estimated CET after overestimation adjustment and the lower bound of CET were 1.45 and 1.16 times of GDP per capita, respectively. Also, the 95% CI of the estimated VSQ was 1.36–1.55 times. The base-case results are presented in Tables 2 and 4.

**Table 2:** Estimates of CET as times of GDP per capita in the base case and using alternative VSL estimates

VSL scenario [references]	VSQ as times of GDP per capital	95% CI
Base case	1.45	1.36–1.55
Yang et al. (2016) [38]	1.30	1.26–1.35
Hammitt and Geng (2019) [39]	1.98	1.58–2.37
Jin et al. (2018) [40]	1.76	1.40–2.14
Hao et al. (2019) [41]	1.23	1.00–1.50
Zheng et al. (2019) [42]	1.08	1.04–1.12

*CI* confidence interval

The VSQ estimates of using VSLs from individual studies instead of weighted mean from the first set of scenario analyses are also presented in Table 2 along with their 95% CIs. The estimated VSQ ranged from 1.08 (95% CI 1.04–1.12) to 1.98 (95% CI 1.73–2.37) times of GDP per capita.

The results of the second set of scenario analyses in which alternative Chinese population utility estimates were used are listed in Table 3 together with the sensitivity analyses of 10% change in utility inputs. When two alternative utility estimates were used, the corresponding estimates of VSQ were 1.25 and 1.20 times of GDP per capita. When the base-case utility inputs varied by 10% upward and downward, the estimated VSQ decreased to 1.33 and increased to 1.61 times of GDP per capita. When the alternative utility estimates were combined with 10% change of inputs, the estimated VSQ varied between 1.14 and 1.39 times of GDP per capita.

**Table 3:** CET estimates using alternative utility sources

Scenario	Utility estimate		
	Base case^a^	Alternative set 1^b^	Alternative set 2^c^
Base case	1.45	1.25	1.20
− 10% utility	1.61	1.39	1.34
+ 10% utility*	1.33	1.16	1.14

*The maximum utility value was limited to 1

^a^Base on data from the National Health Services Survey 2008 [28]

^b^Base on data from Si, Lei et al. [32]

^c^Based on EuroQol study [43]

When the annual discount rates of 3% and 8% were used, the VSQ estimate was 1.15 and 1.93 times of GDP per capita, respectively (Table 4).

**Table 4:** CET estimates using alternative discount rates

Discount rate	VSQ as times of GDP per capita	LB & UB
5% (Base case)	1.45	1.16–2.90
3%	1.15	0.92–2.30
8%	1.93	1.54–3.86

## Discussion

In the present study, we estimated the CET of a QALY in China using the VSL approach proposed by Tehard et al. [12] with overestimation correction. The point estimate, the lower bound, and the upper bound were roughly 1.5, 1.2, and 3.0 times of GDP per capita in China. To our knowledge, this study represents the first initiative to estimate the CET of a QALY in the Chinese setting. Therefore, it fills an important information gap in the evidence base of health policy decision-making in China.

The lower and upper bounds of our estimates resonated with the widely accepted but not necessarily theoretically convincing once and three times of GDP per capita CETs [10], which are also the recommended CETs in the Chinese pharmacoeconomics guidelines [27]. The implications of our results are at least twofold. First, the 1.5 times of GDP per capita CET may be considered as the cut-off if go or no-go decisions are to be made based on QALY-enabled CEAs. Since an explicit cut-off is absent for decision-making in China, industry health economics practitioners may prefer to benchmark the three times the GDP per capita threshold, whereas the public policy makers, which are usually able to leverage the bargaining power, incline to opt for lower but equivocal thresholds to counteract industry practices. Whereas the three times the GDP per capita threshold is not unreasonable, it is more likely to be the upper bound, and should not be a common reference for price-setting. On the other hand, efforts to lower drug prices via negotiations should incorporate an appropriate amount of value protection. For example, over-exploited bargaining power may disincentivize future innovations. Taken together, the 1.5 times GDP per capita can be considered as a reference for decision-making to minimize the chances of suboptimal decisions. Second, the validity of existing CEAs in China that engaged the once and three times of GDP per capita cut-offs is partially supported by the current results as far as the CETs are concerned. To date, the economic evaluations conducted in the Chinese setting most frequently engaged the one and three times the GDP per capita thresholds [35]. Based on the current results, the CETs adopted by the scholarly publications are within the reasonable ranges. At the minimum, the previous research outputs can be used as the best- and worst-case scenarios. In the meantime, future studies may consider to use the CET of 1.5 times the GDP per capita.

Of note, the estimated VSQ stayed above once the GDP per capita in the numerous scenario and sensitivity analyses, which imparted relatively strong robustness to the once the GDP per capita CET as a bottom line. A potential inference is that the CET may not be lower than once the GDP per capita even in conservative settings. It has been argued that healthcare decision-making rarely fits a simple process of using a single CET [36]. From the public policy point of view, conservative choices may be preferred when faced with strong uncertainty due to risk-averse attitudes [37]. At the presence of ambiguity over the CET, decision-makers may choose the bottom-line scenario for prudence. The once the GDP per capita thus serves as a reference to calculate the floor prices.

The present study used the VSL approach to estimate the CET, thereby circumventing the requirement of enduring external healthcare budget shocks in the healthcare system if the CET is to be empirically estimated based on marginal costs of QALY. In addition, we denoted CET as times of GDP per capita throughout the analytic process, dismissing the need of currency inflation and partially mitigating bias due to wealth inequity across regions. The estimation process of correcting the potential overestimation of the original VSL-based approach may represent a marginal novelty of the methodology of CET research, which can be exploited in other countries and regions. Aside from these strengths, several caveats must also be noted when interpreting the results. First, the data of population distribution, the life table, and the VSL data were not necessarily up to date. This is because a full life table instead of an abridged one is mandatory for our research, while the time stamp of population distribution should be kept in line with that of life table. However, the data sources did carry cohesiveness in that the different types of data used in the present study were all collected in or close to 2010. Second, assumptions about the stasis of CET as times of GDP per capita over time and across regions had to be made, yet such assumptions have not been validated in China by far. Third, the present study only relied on the VSL approach to estimate the CET. Due to theoretical and practical challenges, alternative approaches could not be undertaken to confirm the validity of the current estimates.

## Conclusions

There was a lack of evidence on the CET of a QALY in China, which hampered science-based healthcare decisions. The present study took the initiative to provide such estimates using a modified VSL approach allowing correction of overestimation by the conventional approach. According to our findings, the CET of a QALY in China is close to 1.5 times of GDP per capita, whereas the lower and upper bounds are approximately 1.2 and 3.0 times of GDP per capita. With conservative assumptions, the CET is close to but still above once the GDP per capita. The 1.5 times GDP per capita may be considered as the benchmark for ICER-based decisions. In addition, the lower and upper bounds of CET based on the present study partially confirm the validity of the inferences from previously published health economic evaluations in the setting of China. More, the results were robust in a number of alternative scenarios. However, limitations of the estimation including data recency and population representativeness should be noted. Future studies in this area should improve the present estimates using updated data and engaging alternative approaches.

## Supplementary Information

Below is the link to the electronic supplementary material.

Supplementary file1 (DOCX 40 kb)

## Data Availability

Data directly used in the study are available in the article and the supplementary materials. The analytic file has been submitted for review and is available from the corresponding authors upon reasonable requests.
