# Supplementary material for: Estimation of the cost-effective threshold of a quality-adjusted life year in China based on the value of statistical life
Source: Eur J Health Econ. 2021 Oct 16;23(4):607–15. doi: 10.1007/s10198-021-01384-z (PMC9135816; doi:10.1007/s10198-021-01384-z)
Supplement: Supplementary file 1 — Supplementary file1 (DOCX 40 kb) [file 10198_2021_1384_MOESM1_ESM.docx]

Table S1. PubMed Search strategy

| number | Search strategy |
| --- | --- |
| #1 | China[Title/Abstract] |
| #2 | VSL[Title/Abstract] OR value of statistical life[Title/Abstract] |
| #3 | health [Title/Abstract] OR mortality [Title/Abstract] |
| #4 | Willingness-to-pay[Title/Abstract] OR WTP[Title/Abstract] |
| #5 | #3 and #4 |
| #6 | #1 and (#2 or #5) |

Table S2. Age specific life expectancy and population of China

|  |  | Male |  |  |  | Female |  |
| --- | --- | --- | --- | --- | --- | --- | --- |
| Age | Probability of dying | Life expectancy | Population |  | Probability of dying | Life expectancy | Population |
| 0 | 0.01671 | 72.38 | 7461199 |  | 0.01753 | 77.30 | 6325235 |
| 1 | 0.00142 | 72.60 | 8574973 |  | 0.00129 | 77.67 | 7082982 |
| 2 | 0.00082 | 71.70 | 8507697 |  | 0.00070 | 76.77 | 7109678 |
| 3 | 0.00061 | 70.76 | 8272491 |  | 0.00048 | 75.82 | 6978314 |
| 4 | 0.00051 | 69.81 | 8246206 |  | 0.00039 | 74.86 | 6973835 |
| 5 | 0.00045 | 68.84 | 7988151 |  | 0.00035 | 73.89 | 6743986 |
| 6 | 0.00045 | 67.87 | 8034452 |  | 0.00031 | 72.92 | 6770018 |
| 7 | 0.00042 | 66.90 | 7292300 |  | 0.00026 | 71.94 | 6136861 |
| 8 | 0.00042 | 65.93 | 7423559 |  | 0.00026 | 70.96 | 6243397 |
| 9 | 0.00043 | 64.96 | 7726203 |  | 0.00025 | 69.97 | 6522622 |
| 10 | 0.00045 | 63.99 | 7830808 |  | 0.00028 | 68.99 | 6623549 |
| 11 | 0.00043 | 63.01 | 7522558 |  | 0.00027 | 68.01 | 6413156 |
| 12 | 0.00045 | 62.04 | 8288987 |  | 0.00027 | 67.03 | 7110572 |
| 13 | 0.00044 | 61.07 | 8161000 |  | 0.00026 | 66.05 | 7064032 |
| 14 | 0.00047 | 60.10 | 8463924 |  | 0.00026 | 65.07 | 7429876 |
| 15 | 0.00055 | 59.12 | 9524898 |  | 0.00028 | 64.08 | 8499586 |
| 16 | 0.00057 | 58.16 | 9795181 |  | 0.00028 | 63.10 | 8995340 |
| 17 | 0.00064 | 57.19 | 10760828 |  | 0.00031 | 62.12 | 10014541 |
| 18 | 0.00068 | 56.23 | 10744556 |  | 0.00032 | 61.14 | 10010718 |
| 19 | 0.00073 | 55.26 | 11079367 |  | 0.00031 | 60.16 | 10464099 |
| 20 | 0.00080 | 54.30 | 14201091 |  | 0.00034 | 59.17 | 13825863 |
| 21 | 0.00081 | 53.35 | 13357755 |  | 0.00035 | 58.19 | 13198894 |
| 22 | 0.00084 | 52.39 | 12281148 |  | 0.00037 | 57.21 | 12193044 |
| 23 | 0.00092 | 51.43 | 12876542 |  | 0.00040 | 56.24 | 12819413 |
| 24 | 0.00097 | 50.48 | 11292037 |  | 0.00041 | 55.26 | 11366731 |
| 25 | 0.00100 | 49.53 | 9969984 |  | 0.00043 | 54.28 | 9963699 |
| 26 | 0.00098 | 48.58 | 9879292 |  | 0.00042 | 53.30 | 9829885 |
| 27 | 0.00100 | 47.62 | 9801611 |  | 0.00045 | 52.33 | 9679225 |
| 28 | 0.00105 | 46.67 | 11271599 |  | 0.00044 | 51.35 | 11050548 |
| 29 | 0.00116 | 45.72 | 9914552 |  | 0.00051 | 50.37 | 9653457 |
| 30 | 0.00116 | 44.77 | 9604727 |  | 0.00054 | 49.40 | 9323642 |
| 31 | 0.00129 | 43.82 | 10141582 |  | 0.00058 | 48.42 | 9724876 |
| 32 | 0.00137 | 42.88 | 9909833 |  | 0.00059 | 47.45 | 9565041 |
|  |  | Male |  |  |  | Female |  |
| Age | Probability of dying | Life expectancy | Population |  | Probability of dying | Life expectancy | Population |
| 33 | 0.00140 | 41.94 | 9289224 |  | 0.00061 | 46.48 | 8890254 |
| 34 | 0.00156 | 41.00 | 10576456 |  | 0.00073 | 45.51 | 10112568 |
| 35 | 0.00174 | 40.06 | 10817432 |  | 0.00076 | 44.54 | 10369084 |
| 36 | 0.00178 | 39.13 | 11690644 |  | 0.00081 | 43.57 | 11216336 |
| 37 | 0.00190 | 38.20 | 12283353 |  | 0.00086 | 42.61 | 11706855 |
| 38 | 0.00201 | 37.27 | 12662559 |  | 0.00092 | 41.64 | 12067901 |
| 39 | 0.00225 | 36.34 | 12937116 |  | 0.00101 | 40.68 | 12274679 |
| 40 | 0.00249 | 35.42 | 13993123 |  | 0.00118 | 39.72 | 13404096 |
| 41 | 0.00257 | 34.51 | 12723691 |  | 0.00120 | 38.77 | 12232606 |
| 42 | 0.00302 | 33.60 | 13782610 |  | 0.00141 | 37.81 | 13249932 |
| 43 | 0.00314 | 32.70 | 10856214 |  | 0.00145 | 36.87 | 10499534 |
| 44 | 0.00339 | 31.80 | 12253040 |  | 0.00162 | 35.92 | 11759118 |
| 45 | 0.00380 | 30.91 | 12252515 |  | 0.00183 | 34.98 | 11710059 |
| 46 | 0.00389 | 30.02 | 11867147 |  | 0.00185 | 34.04 | 11488631 |
| 47 | 0.00414 | 29.14 | 13803796 |  | 0.00201 | 33.10 | 13168361 |
| 48 | 0.00512 | 28.26 | 10224798 |  | 0.00246 | 32.17 | 9850286 |
| 49 | 0.00540 | 27.40 | 5628162 |  | 0.00260 | 31.25 | 5600798 |
| 50 | 0.00586 | 26.55 | 7205176 |  | 0.00295 | 30.33 | 6891832 |
| 51 | 0.00601 | 25.70 | 6624865 |  | 0.00308 | 29.42 | 6213967 |
| 52 | 0.00639 | 24.85 | 8570000 |  | 0.00323 | 28.50 | 8047709 |
| 53 | 0.00708 | 24.01 | 9422827 |  | 0.00363 | 27.60 | 8929153 |
| 54 | 0.00795 | 23.18 | 8540366 |  | 0.00417 | 26.69 | 8307276 |
| 55 | 0.00825 | 22.36 | 8973192 |  | 0.00433 | 25.80 | 8637336 |
| 56 | 0.00898 | 21.54 | 8981235 |  | 0.00474 | 24.91 | 8756892 |
| 57 | 0.00966 | 20.73 | 8099033 |  | 0.00517 | 24.03 | 7994855 |
| 58 | 0.01080 | 19.93 | 8153588 |  | 0.00578 | 23.15 | 8014345 |
| 59 | 0.01205 | 19.14 | 6875890 |  | 0.00659 | 22.28 | 6826108 |
| 60 | 0.01323 | 18.37 | 6917026 |  | 0.00743 | 21.43 | 6701178 |
| 61 | 0.01454 | 17.61 | 6690003 |  | 0.00818 | 20.59 | 6339122 |
| 62 | 0.01593 | 16.86 | 5719180 |  | 0.00921 | 19.75 | 5557673 |
| 63 | 0.01700 | 16.12 | 5492805 |  | 0.00995 | 18.93 | 5298828 |
| 64 | 0.01982 | 15.39 | 5015412 |  | 0.01174 | 18.11 | 4936055 |
| 65 | 0.02141 | 14.70 | 4564266 |  | 0.01300 | 17.32 | 4509145 |
| 66 | 0.02218 | 14.01 | 4391409 |  | 0.01347 | 16.55 | 4249556 |
|  |  | Male |  |  |  | Female |  |
| Age | Probability of dying | Life expectancy | Population |  | Probability of dying | Life expectancy | Population |
| 67 | 0.02574 | 13.31 | 4003493 |  | 0.01588 | 15.76 | 3938648 |
| 68 | 0.02781 | 12.65 | 3904424 |  | 0.01725 | 15.01 | 3836444 |
| 69 | 0.03242 | 12.00 | 3884879 |  | 0.02040 | 14.27 | 3831018 |
| 70 | 0.03760 | 11.38 | 3724605 |  | 0.02401 | 13.55 | 3664807 |
| 71 | 0.03880 | 10.81 | 3116177 |  | 0.02558 | 12.87 | 3149541 |
| 72 | 0.04468 | 10.22 | 3449237 |  | 0.02965 | 12.20 | 3443988 |
| 73 | 0.04838 | 9.68 | 3149307 |  | 0.03243 | 11.56 | 3194562 |
| 74 | 0.05405 | 9.15 | 2964127 |  | 0.03614 | 10.93 | 3116046 |
| 75 | 0.06000 | 8.64 | 2690547 |  | 0.03999 | 10.32 | 2941930 |
| 76 | 0.06005 | 8.16 | 2454168 |  | 0.04141 | 9.73 | 2721332 |
| 77 | 0.07205 | 7.65 | 2420196 |  | 0.05015 | 9.13 | 2662187 |
| 78 | 0.07875 | 7.20 | 1983724 |  | 0.05564 | 8.58 | 2271134 |
| 79 | 0.08580 | 6.78 | 1730224 |  | 0.06228 | 8.06 | 1976691 |
| 80 | 0.10143 | 6.37 | 1716514 |  | 0.07475 | 7.56 | 2020745 |
| 81 | 0.10571 | 6.03 | 1257795 |  | 0.07911 | 7.13 | 1558898 |
| 82 | 0.11501 | 5.68 | 1212683 |  | 0.08774 | 6.70 | 1545235 |
| 83 | 0.12435 | 5.36 | 964710 |  | 0.09592 | 6.29 | 1272428 |
| 84 | 0.13712 | 5.05 | 765800 |  | 0.10625 | 5.91 | 1058390 |
| 85 | 0.14642 | 4.77 | 672819 |  | 0.11380 | 5.55 | 975341 |
| 86 | 0.15616 | 4.50 | 530641 |  | 0.12207 | 5.20 | 813574 |
| 87 | 0.16737 | 4.24 | 408984 |  | 0.13457 | 4.85 | 656292 |
| 88 | 0.18201 | 3.99 | 324282 |  | 0.14958 | 4.53 | 534597 |
| 89 | 0.19847 | 3.77 | 263084 |  | 0.16115 | 4.24 | 452314 |
| 90 | 0.21954 | 3.58 | 193982 |  | 0.18062 | 3.96 | 359823 |
| 91 | 0.22364 | 3.45 | 126484 |  | 0.19240 | 3.72 | 244595 |
| 92 | 0.23992 | 3.29 | 94157 |  | 0.20920 | 3.49 | 193519 |
| 93 | 0.23884 | 3.18 | 66717 |  | 0.21750 | 3.28 | 142574 |
| 94 | 0.23810 | 3.02 | 49532 |  | 0.22023 | 3.05 | 106924 |
| 95 | 0.24132 | 2.80 | 36268 |  | 0.23402 | 2.78 | 81254 |
| 96 | 0.23725 | 2.53 | 28664 |  | 0.23725 | 2.47 | 62225 |
| 97 | 0.20551 | 2.17 | 22045 |  | 0.22992 | 2.08 | 46603 |
| 98 | 0.19739 | 1.60 | 18355 |  | 0.22438 | 1.56 | 36334 |
| 99 | 0.26848 | 0.87 | 12384 |  | 0.27175 | 0.86 | 25847 |

Table S3. Utility scenarios for estimating VSQ/GDP per capita

| Base case^a^ | |  | Scenario 1^b^ | |  | Scenario 2^c^ | |
| --- | --- | --- | --- | --- | --- | --- | --- |
| Age-specific | Utility |  | Age-specific | Utility |  | Age-specific | Utility |
| 15–19 | 0.897 |  | 18-24 | 0.956 |  | 18-24 | 0.990 |
| 20–24 | 0.885 |  | 25-29 | 0.954 |  | 25-34 | 0.980 |
| 25–29 | 0.873 |  | 30-34 | 0.949 |  | 35-44 | 0.970 |
| 30–34 | 0.854 |  | 35-39 | 0.944 |  | 45-54 | 0.960 |
| 35–39 | 0.840 |  | 40-44 | 0.937 |  | 55-64 | 0.930 |
| 40–44 | 0.825 |  | 45-49 | 0.936 |  | 65-74 | 0.900 |
| 45–49 | 0.803 |  | 50-54 | 0.929 |  | 75+ | 0.840 |
| 50–54 | 0.783 |  | 55-59 | 0.911 |  |  |  |
| 55–59 | 0.763 |  | 60-64 | 0.886 |  |  |  |
| 60–64 | 0.740 |  | 65-69 | 0.878 |  |  |  |
| 65–69 | 0.714 |  | 70-74 | 0.842 |  |  |  |
| 70–74 | 0.693 |  | 75-79 | 0.833 |  |  |  |
| 75–79 | 0.677 |  | 80-84 | 0.778 |  |  |  |
| 80–84 | 0.659 |  | 85+ | 0.683 |  |  |  |
| 85–89 | 0.652 |  |  |  |  |  |  |

^a^ Base on data from the National Health Services Survey 2008 (Sun et al., 2011).

^b^ Base on data from Si, Lei et al. (Si et al., 2017).

^c^ Based on EuroQol study (Szende A, 2014).

References

Si, L., Shi, L., Chen, M., & Palmer, A.J. (2017). Establishing benchmark EQ-5D-3L population health state utilities and identifying their correlates in Gansu Province, China. *Qual Life Res,* 26, 3049-3058.

Sun, S., Chen, J., Johannesson, M., Kind, P., Xu, L., Zhang, Y., et al. (2011). Population health status in China: EQ-5D results, by age, sex and socio-economic status, from the National Health Services Survey 2008. *Quality of life research : an international journal of quality of life aspects of treatment, care and rehabilitation,* 20, 309-320.

Szende A, J.B., Cabases J, editors (2014). *Self-Reported Population Health: An International Perspective based on EQ-5D*. Dordrecht (NL): Springer.
